# Retraction Note to: lncRNA HAND2-AS1 is downregulated in osteoarthritis and regulates IL-6 expression in chondrocytes

**DOI:** 10.1186/s13018-022-03206-1

**Published:** 2022-06-14

**Authors:** Zhenxing Si, Shifeng Zhou, Zilong Shen, Feiyu Luan, Jinglong Yan

**Affiliations:** 1 Orthopedic Department, Second Affiliated Hospital of Harbin Medical University, No. 148 Baojian Road, Nangang District, Harbin, Heilongjiang China; 2 Surgical Emergency, First Affiliated Hospital of Harbin Medical University, Harbin, China

## Retraction Note: Journal of Orthopaedic Surgery and Research (2021) 16:68 10.1186/s13018-021-02216-9

The Editor-in-Chief has retracted this article. After publication, the authors contacted the publisher to request a retraction because a wrong Trial Registration Number was used. After further investigation, concerns were raised about the western blots presented in the article. The authors did not supply raw data for these, or evidence of ethics approval on request. Therefore, the Editor-In-Chief no longer has confidence in the integrity of the data in this article.

Authors have not responded to any correspondence from the editor about this retraction.

